# Planned versus unplanned rotational atherectomy for plaque modification in severely calcified coronary lesions

**DOI:** 10.1007/s00392-023-02176-6

**Published:** 2023-03-17

**Authors:** Lucas Bacmeister, Philipp Breitbart, Karolina Sobolewska, Klaus Kaier, Faridun Rahimi, Nikolaus Löffelhardt, Christian Valina, Franz-Josef Neumann, Dirk Westermann, Miroslaw Ferenc

**Affiliations:** 1 Department of Cardiology and Angiology, Medical Center - University of Freiburg, Faculty of Medicine, University of Freiburg, Südring 15, 79189 Bad Krozingen, Germany; 2 Institute of Medical Biometry and Statistics, Faculty of Medicine and Medical Center, University of Freiburg, Freiburg, Germany

**Keywords:** Percutaneous coronary intervention, Severe calcification, Rotational atherectomy, Plaque modification, Unplanned procedures

## Abstract

**Background:**

Evidence on the optimal timing of RA is scarce, although increased periprocedural complications for unplanned procedures have been reported.

**Aims:**

To compare planned versus unplanned use of rotational atherectomy (RA) for plaque modification in patients with severely calcified coronary lesions.

**Methods:**

Procedural and 1-year follow-up data of planned (*n = *562 lesions in 448 vessels of 416 patients) and unplanned (*n = *490 lesions in 435 vessels of 403 patients) RA between 2008 and 2020 were analyzed using the propensity score methods. The primary composite endpoint was target lesion failure (TLF), defined as cardiovascular death (CVD), target vessel myocardial infarction (TVMI), or target lesion revascularization (TLR).

**Results:**

Angiographic success was > 99% in both groups. Fluoroscopy time and contrast volume were significantly lower in planned RA (*p < *0.001). Periprocedural complications including slow-flow, coronary dissection, and MI occurred in 4.8% after planned, and in 5.7% after unplanned RA. TLF occurred in 18.5% after planned, and in 14.7% after unplanned RA. Weighted subdistribution hazard ratios for TLFs revealed an unfavorable 1-year outcome for planned RA (sHR 1.62 [1.07–2.45], *p = *0.023), which was driven by TLR (sHR 2.01 [1.18–3.46], *p = *0.011), but not by CVD, or TVMI. No differences were observed in all-cause mortality.

**Conclusions:**

Unplanned RA was associated with favorable outcome when compared to planned RA. Thus, RA can safely be reserved for lesions that prove untreatable by conventional means. Randomized and prospective trials are needed to evaluate a predominant use of rotational atherectomy as a bailout strategy in the future.

**Graphical abstract:**

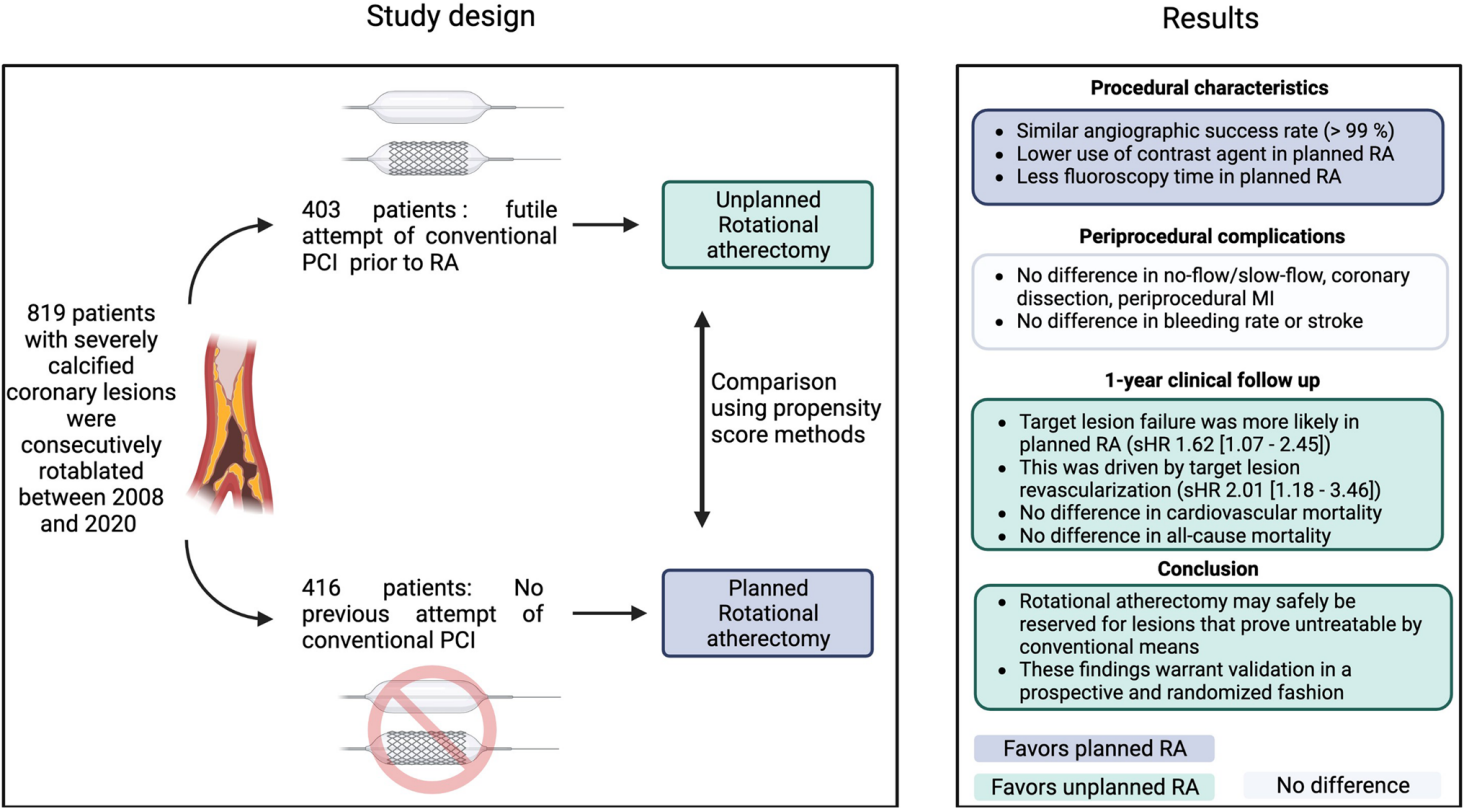

**Supplementary Information:**

The online version contains supplementary material available at 10.1007/s00392-023-02176-6.

## Introduction

The extent of coronary artery calcification depends on age and is more common in male than in female patients [1]. Especially in the light of an aging population, the prevalence of calcified coronary lesions in patients with coronary artery disease (CAD) is steadily increasing. Angiographic data suggest that up to one-third of coronary lesions can be classified as moderately to severely calcified, resulting in a higher risk of impossible balloon crossing, stent underexpansion and disruption of antiproliferative stent polymer coating, subsequently increasing the rate of target lesion revascularization and restenosis [2–4].

Rotational atherectomy (RA) was developed in the late 1980s [5] and became the most used plaque modification technique in the era of drug-eluting stents (DES). Results from ROTAXUS have questioned routine use of RA in heavily calcified lesions [6], although data from PREPARE-CALC showed superior strategy success compared with scoring or cutting balloons [7]. Hence, the role of RA in interventional cardiology is contemporarily interpreted as an unplanned (or ‘provisional’) strategy upon an upfront device failure, or as the initial, planned procedure, when angiography or intravascular imaging reveal severe calcifications [8].

Evidence whether planned or unplanned RA has favorable outcome is limited. Previous studies found lower procedural time and contrast volume when RA was done as a planned procedure [9–13]. However, data on periprocedural complications and post-discharge outcomes are conflicting in these studies [9–13]. Therefore, we aimed to add evidence to this ongoing debate with a comparison of procedural and 1-year outcomes of planned vs. unplanned use of RA.

## Methods

### Study design and patient population

All patients who received RA as lesion preparation for severely calcified coronary lesions at our center between March 2008 and March 2020 were included in this retrospective analysis. RA was adjudicated as “planned”, when rotational atherectomy was the initial strategy for lesion preparation, or as “unplanned” strategy in the case of prior device failure (e.g., impossible balloon crossing, balloon expansion failure, or failed stent delivery). Indication for planned RA was at the choice of an experienced operator and was based on angiographic findings such as grade of calcification, lesion length, lesion angulation, lesion tortuosity, and lesion eccentricity.

The procedures were performed by experienced interventionalists with a history of > 150 coronary rotational atherectomies. Importantly, planned and unplanned RA were performed by the same interventionalists. All patients gave written informed consent for the pseudonymized use of clinical, procedural, and follow-up data at the time of the intervention. The study was approved by the local institutional review board (EK 21-1100) and complies with the Declaration of Helsinki.

### Procedural characteristics

The Rotablator™ or the ROTAPRO™ (both Boston Scientific Scimed, Inc., USA) were used for RA. Burr/vessel ratios of 0.4–0.6 were aspired and a maximum ratio of 0.7 was tolerated. In accordance with current recommendations [14], rotational speed ranged between 130.000 and 165.000 rpm, decelerations of > 5.000 rpm were prevented, a continuous for- and backward shift was applied (“pecking”) and burr time was kept as short as possible. Prior to RA, all patients received 500 mg aspirin and a loading dose of an oral P2Y12-receptor antagonist (either clopidogrel, prasugrel, or ticagrelor). Duration and use of antiplatelet agents were recommended according to the ESC-guidelines valid at the time of intervention. This predominantly depended on (i) presentation with acute- or chronic coronary syndrome, and (ii) on individual bleeding and ischemia risk.

### Endpoints

The primary composite endpoint was target lesion failure (TLF), defined as cardiovascular death (CVD), target vessel myocardial infarction (MI), and/or target lesion revascularization (TLR). Certain and suspected cardiac death were defined as CVD. MI was diagnosed by clinical symptoms, electrocardiography, and elevated cardiac biomarkers in accordance with the fourth universal definition of myocardial infarction [15]. TLR was defined as any repeated revascularization in a range of ± 5 mm of the initially deployed stent, either by percutaneous coronary intervention (PCI) or coronary artery bypass grafting (CABG). Secondary endpoints included components of the primary endpoint as well as procedural and fluoroscopy time, contrast volume, angiographic success, and incidence of periprocedural complications. In unplanned RA, fluoroscopy time and the use of contrast volume of a prior failed attempt of conventional PCI were included into analysis. Angiographic success was defined as a residual stenosis of < 30% or grade 3 poststenotic coronary flow according to the Thrombolysis in Myocardial Infarction (TIMI) classification. Periprocedural complications included slow-flow/low-flow, periprocedural MI, and coronary dissection. The endpoints TLF, TLR, and TV-MI were analyzed per vessel, CVD and all-cause mortality were analyzed per patient. Multiple interventions in different lesions within the same coronary vessel (RCA, LCX, or LAD) were subsumed under one vessel at risk. If the LM was involved, solely lesions interventions of lesions in the right coronary system were included as an additional vessel at risk. In line with this, for the analyses of TLF, TLR, and TVMI the maximum number of vessels at risk per patient was 2.

As part of our clinical routine, follow-ups were performed by structured telephone interviews with patients or their general practitioners by assessors blinded to patient and procedural characteristics. For the adjudication of procedural und periprocedural outcomes, as well as angiographic endpoints (TVMI and target lesion revascularization), digitally recorded coronary angiographies of patients undergoing RA between 2008 and 2020 were retrospectively assessed offline by investigators blinded to the clinical outcomes of these patients.

### Statistics

Statistical analysis was performed using the SPSS software, Version 25 (IBM Corp., Armonk, NY, USA) and Stata 17 (StataCorp, College Station, Texas, USA). Categorical data are depicted as frequencies and percentages, continuous variables as mean with standard deviation or median with interquartile range. Chi-square testing for binary variables and students *t* tests or Wilcoxon–Mann–Whitney tests for continuous variables were performed.

Unadjusted cumulative incidences were calculated using the Kaplan–Meier Method (all-cause mortality) or the Aalen Johansen estimator (all other endpoints) with “death” as competing event. For the analysis of all-cause mortality, hazard ratios were estimated using the cox model. For all other endpoints and subdistribution hazard ratios were estimated using the Fine-Gray model with all-cause mortality as competing event. All regression models were estimated on the level of the vessel (rather than the patient level) with robust standard errors to take clustering within patients into account. Since patients were not randomized towards planned or unplanned RA, potential confounding factors were taken into account using the propensity score methods. Thereby, inverse probability weighting was applied. The propensity score is defined as the conditional probability of an individual for being in the treatment group, given a group of observed covariates. For the propensity score estimations, we fit a logistic regression model with all characteristics listed in Tables 1, 2 and 3 as independent variables. We checked the balance of covariates by inspection of balancing statistics (the standardized mean difference of covariate distribution between treatment groups). After weighting, all regression models were re-estimated and (weighted) cumulative incidences were predicted based on the respective regression model. Two-sided *p* values are given, and statistical significance was considered as *p* value < 0.05. No adjustments for multiple testing were done.

**Table 1:** Baseline characteristics of the study cohort

	Planned RA	Unplanned RA	*p* value
	*n = *416 patients	*n = *403 patients	
Age (years)	74.2 ± 8.9	71.1 ± 8.9	< 0.001
Male (%)	79.1	79.7	0.863
Cardiovascular risk factors			
Hypertension (%)	92.3	92.3	1
Diabetes (%)	35.8	43.7	0.022
Smoking (%)	8.2	10.7	0.233
BMI (kg/m^2^)	27.6 ± 4.4	28.1 ± 4.3	0.058
Family history of premature CAD (%)	29.8	31.5	0.649
Laboratory findings			
LDL-cholesterol (mg/dl)	97 ± 38	96 ± 35	0.687
Creatinine (mg/dl)	1.3 ± 1.0	1.2 ± 0.8	0.155
CRP (mg/dl)	0.9 ± 2.8	0.8 ± 1.8	0.53
Hemoglobin (g/dl)	13.4 ± 1.8	13.5 ± 1.8	0.395
Thrombocyte count (*1.000/µl)	219 ± 75	220 ± 66	0.84
Clinical history			
Prior MI	28.4	39.2	0.001
Prior PCI (%)	51.7	59.6	0.025
Prior CABG (%)	26.4	30.3	0.245
LVEF (%)	51.7 ± 11.7	51.9 ± 11.0	0.823
Statin use > 5 years (%)	24.8	21.3	0.281

Data presented as mean ± standard deviation or percentage

*ACS* acute coronary syndrome, *BMI* body mass index, *CABG* coronary artery bypass grafting, *CAD* coronary artery disease, *CRP* C-reactive protein, *LDL* low-density lipoprotein, *LVEF* left ventricular ejection fraction, *MI* myocardial infarction, *PCI* percutaneous coronary intervention

**Table 2:** Angiographic characteristics of the study cohort

	Planned RA	Unplanned RA	*p* value
	*n = *562 lesions	*n = *490 lesions	
Coronary artery disease [*n* (%)]			0.009
1-vessel CAD	25 (4.4)	43 (8.8)	
2-vessel CAD	99 (17.6)	95 (19.4)	
3-vessel CAD	438 (77.9)	352 (71.8)	
Target lesion [*n* (%)]			< 0.001
Left main	81 (14.4)	20 (4.1)	
Left anterior descending	213 (38.0)	142 (29.0)	
Left circumflex	111 (19.8)	123 (25.1)	
Right coronary artery	154 (27.5)	203 (41.4)	
Coronary bypass vessel	3 (0.5)	2 (0.4)	
Grade of calcification [*n* (%)]			0.013
Moderate	44 (7.8)	61 (12,4)	
Severe	518 (92.2)	429 (87.6)	
Angulation of the lesion [n (%)]			0.027
Mild	247 (44.0)	178 (36.3)	
Moderate	285 (50.7)	275 (56.1)	
Severe	30 (5.3)	37 (7.6)	
Lesion length [*n* (%)]			0.585
< 10 mm	31 (5.5)	26 (5.3)	
10–20 mm	181 (32.2)	144 (29.4)	
> 20 mm	350 (62.3)	320 (65.3)	
Specification of intervened lesions			
Ostial lesions [*n* (%)]	33 (5.9)	27 (5.5)	0.894
Eccentric lesions [*n* (%)]	370 (65.8)	336 (68.6)	0.358
Tortuous lesions [*n* (%)]	131 (23.4)	133 (27.1)	0.176
Chronic total occlusions [*n* (%)]	144 (25.6)	193 (39.4)	< 0.001

Data presented as mean ± standard deviation or percentage

*CAD* coronary artery disease

**Table 3:** Procedural characteristics of the study cohort

	Planned RA	Unplanned RA	*p* value
	*n = *562 lesions	*n = *490 lesions	
Lesions treated [*n* (%)]			< 0.001
1	255 (45.4)	314 (64.1)	
2	273 (48.6)	165 (33.7)	
3	34 (6.0)	11 (2.2)	
Device [*n* (%)]			< 0.001
Rotablator™	307 (54.6)	386 (78.8)	
ROTAPRO™	255 (45.4)	103 (21.0)	
Burr size [*n* (%)]			< 0.001
1.25 mm	70 (12.5)	140 (28.6)	
1.5 mm	274 (48.8)	266 (54.3)	
1.75 mm	187 (33.3)	74 (15.1)	
2.00 mm	30 (5.3)	10 (2.0)	
2.15 mm	1 (0.2)	0 (0.0)	
Guiding catheter [*n* (%)]			< 0.001
6F	152 (27.0)	216 (44.1)	
7F	351 (62.5)	248 (50.6)	
8F	59 (10.5)	26 (5.3)	
Percutaneous coronary intervention			
Balloon diameter (mm)	3.0 (± 1.0)	2.9 (± 2.3)	0.228
Peak inflation pressure (atm)	20.4 (± 4.3)	22.1 (± 7.3)	< 0.001
Max. stent diameter (mm)	3.4 (± 0.53)	3.2 (± 0.54)	< 0.001
Peak pressure at stent delivery (atm)	17.9 (± 4.4)	18.0 (± 5.0)	0.898
Stent length (mm)	43.3 (± 25.7)	48.8 (± 29.2)	< 0.001
Use of an OPN-balloon [*n* (%)]	53 (9.4)	101 (20.6)	< 0.001
Number of stents implanted [*n* (%)]	3.15 (± 1.5)	2.8 (± 1.4)	< 0.001
Stents [*n* (%)]			0.072
No stent	4 (0.7)	3 (0.6)	
BMS	4 (0.7)	5 (1.0)	
1st-generation DES	48 (8.5)	66 (13.5)	
2nd-generation DES	506 (90.0)	416 (84.9)	
Stent polymer coating [*n* (%)]			< 0.001
Paclitaxel	2 (0.4)	4 (0.8)	
Sirolimus	18 (3.2)	19 (3.8)	
Everolimus	357 (63.5)	373 (76.1)	
Zotarolimus	177 (31.5)	86 (17.6)	
Angiographic success [*n* (%)]	557 (99.1)	485 (99.0)	1
Radiation exposure (µGym^2^)	8413 (± 7879)	13,284 (± 13,554)	< 0.001
Fluoroscopy time (min)	34 (± 23)	48 (± 38)	< 0.001
Contrast volume (ml)	228 (± 127)	284 (± 217)	< 0.001

Data presented as mean ± standard deviation or percentage

*DES* drug-eluting stent, *OPN-balloon* very high pressure non-compliant balloon

## Results

**Fig. 1:**
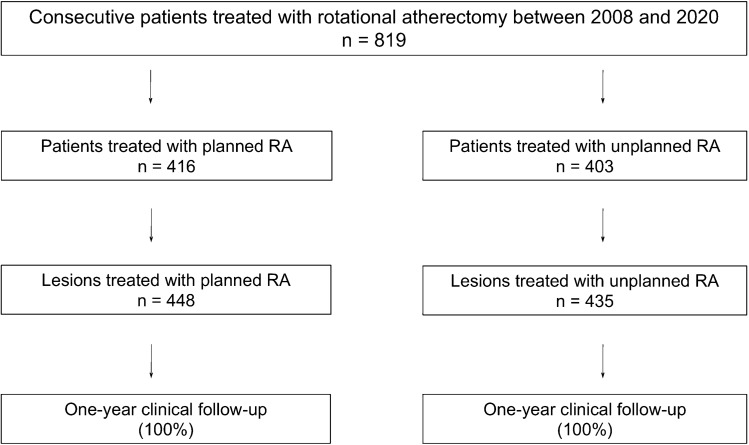
Distribution of planned and unplanned procedures in this study. 819 patients were included into analyses. In 416 patients, rotational atherectomy prior to percutaneous coronary intervention (RA) was done in a planned manner. A total of 562 lesions in 448 vessels were treated in this group. In 403 patients, unplanned RA was performed and 490 lesions in 435 vessels were intervened. A complete clinical 1-year follow-up could be obtained for both groups

We included 819 patients with planned (*n = *416) or unplanned (*n = *403) RA in our study (Fig. 1). 562 lesions in 448 vessels were treated in the planned group and 490 lesions in 435 vessels in the unplanned group. A complete 1-year follow-up could be obtained.

### Patient characteristics

Mean age was 74.2 ± 8.9 years in the planned and 71.1 ± 8.9 years in the unplanned group (Table 1). Apart from a higher prevalence of diabetes in the unplanned group (35.8% vs. 43.7%, *p = *0.022), cardiovascular risk factors did not differ between the groups. Previous PCI (51.7% vs. 59.6%, *p = *0.025) and prior MI (28.4% vs. 39.2%, *p < *0.001) were less prevalent in the planned group. The rates of previous coronary bypass grafting (26.4% vs. 30.3%, *p = *0.245), intervention in the setting of acute coronary syndrome (17.8% vs. 21.3%, *p = *0.217), and left ventricular ejection fraction (51.7% vs. 51.9%, *p = *0.823) were comparable between groups.

### Angiographic characteristics

Coronary angiography identified three-vessel coronary artery disease (CAD) as most prevalent in both groups (77.9% vs. 71.8%) (Table 2). Target lesions of planned interventions were more often the left main (14.4% vs. 4.1%) and the left anterior descending (38.0% vs. 29.0%) coronary arteries. Interventions were predominantly performed in severely calcified lesions, especially planned procedures (92.2% vs. 87.6%, *p = *0.013). The relative number of ostial stenosis, lesion length, and eccentricity and tortuosity of the intervened vessel were comparable between the groups. Chronic total occlusions were less prevalent in the planned group (25.6% vs. 39.4%, *p < *0.001).

### Procedural characteristics

A burr size in the range of 1.25 mm and 1.75 mm and sheath sizes of 6–7 French were utilized for most RAs (Table 3). In 54.6% of planned Ras, more than one lesion was treated, whereas singular interventions were performed in 64.1% of unplanned RAs. In line, significantly more stents per procedure were implanted in the planned group (3.15 ± 1.5 vs. 2.8 ± 1.4, *p < *0.001).

Angiographic success was obtained in most cases and did not differ between the groups (99.1% vs. 99.0%, *p = *1.0). However, fluoroscopy time was significantly longer in the unplanned group (34 ± 23 min vs. 48 ± 38 min, *p < *0.001) and was accompanied by higher radiation exposure (8413 ± 7879 µGym^2^ vs. 13,284 ± 13,554 µGym^2^, *p < *0.001) and higher use of contrast volume (228 ± 127 ml vs. 284 ± 217 ml, *p < *0.001).

### In-hospital outcomes

Periprocedural complications occurred in 4.8% of the patients with planned and in 5.7% of the patients with unplanned procedures (*p = *0.764) (Table 4). Postprocedural complications such as bleedings requiring transfusion or stroke were rare and did not differ between the groups.

**Table 4:** In-hospital outcomes of the study cohort

	Planned RA	Unplanned RA	*p* value
	*n = *416 patients	*n = *403 patients	
Periprocedural complications [*n* (%)]	20 (4.8)	23 (5.7)	0.764
No-flow/slow-flow, dissection periprocedural MI	6 (1.4)	11 (2.7)	
Perforation	10 (2.4)	9 (2.2)	
Emergency surgery	1 (0.2)	1 (0.2)	
Use of Impella / ECLS	3 (0.7)	2 (0.5)	
Postprocedural complications [*n* (%)]			
Transfusions	11 (2.6)	14 (3.5)	0.546
Stroke	2 (0.5)	1 (0.2)	1

Data presented as mean ± standard deviation or percentage

*MI* myocardial infarction, *ECLS* extracorporeal life support system

### Clinical 1-year follow-up

**Fig. 2:**
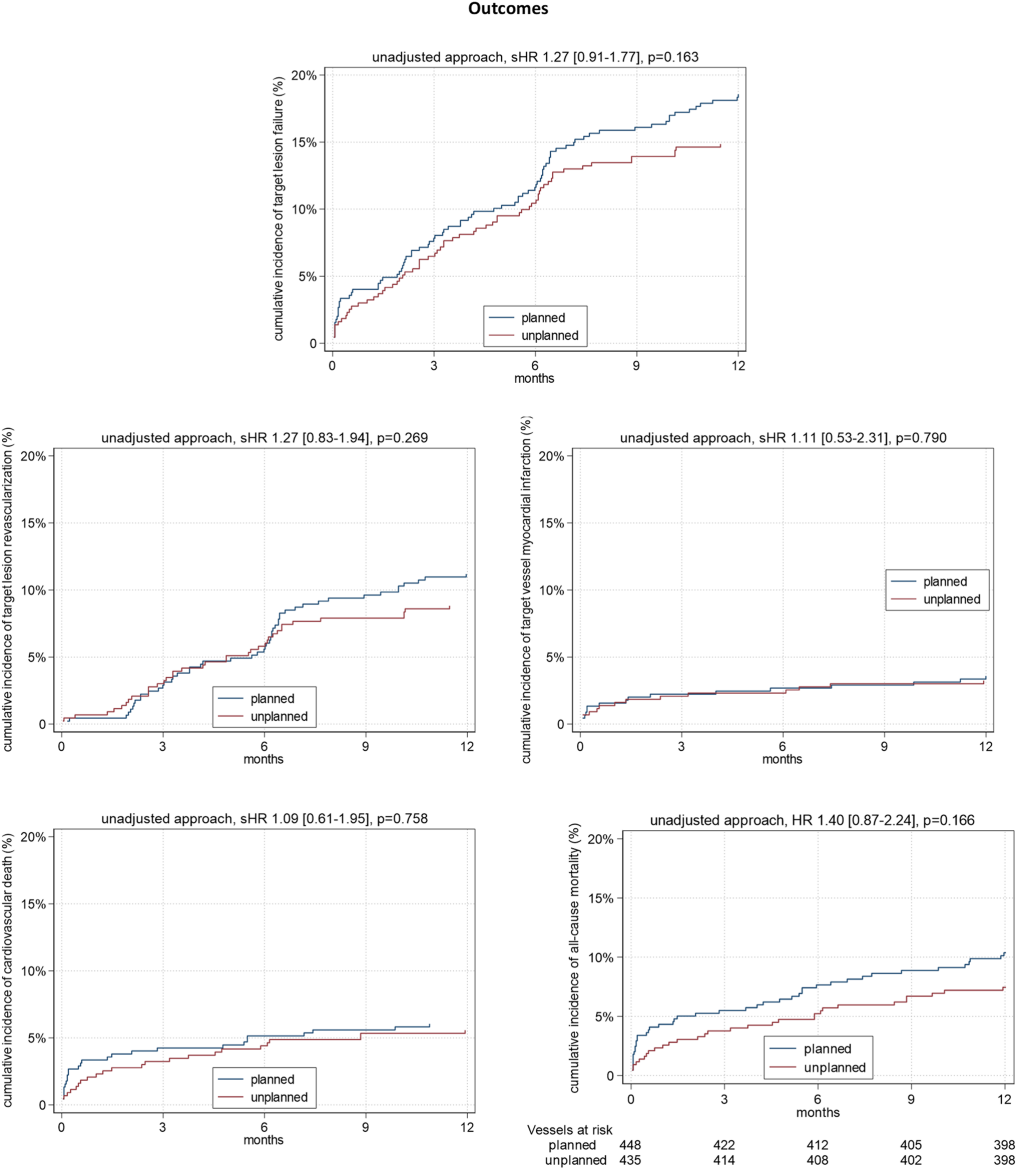
Unadjusted 1-year outcomes after planned versus unplanned rotational atherectomy. Unadjusted cumulative incidences of the composite endpoint target lesion failure comprising target lesion revascularization, target vessel myocardial infarction, cardiovascular death and all-cause mortality for planned (blue) or unplanned (red) rotational atherectomy. Please note that the 883 vessels refer to 818 patients. In case of multiple interventions in different vessels of the same patient, many cases were conducted within the same date (*N = *32) or using the same technique (either planned or unplanned RA in both vessels, *N = *58). Cases with multiple interventions at different dates (*N = *33) or using different techniques (planned or unplanned RA, *N = *17) are also existent and making the analysis of all endpoints on the level of the vessel (rather than the patient level) eminent

**Fig. 3:**
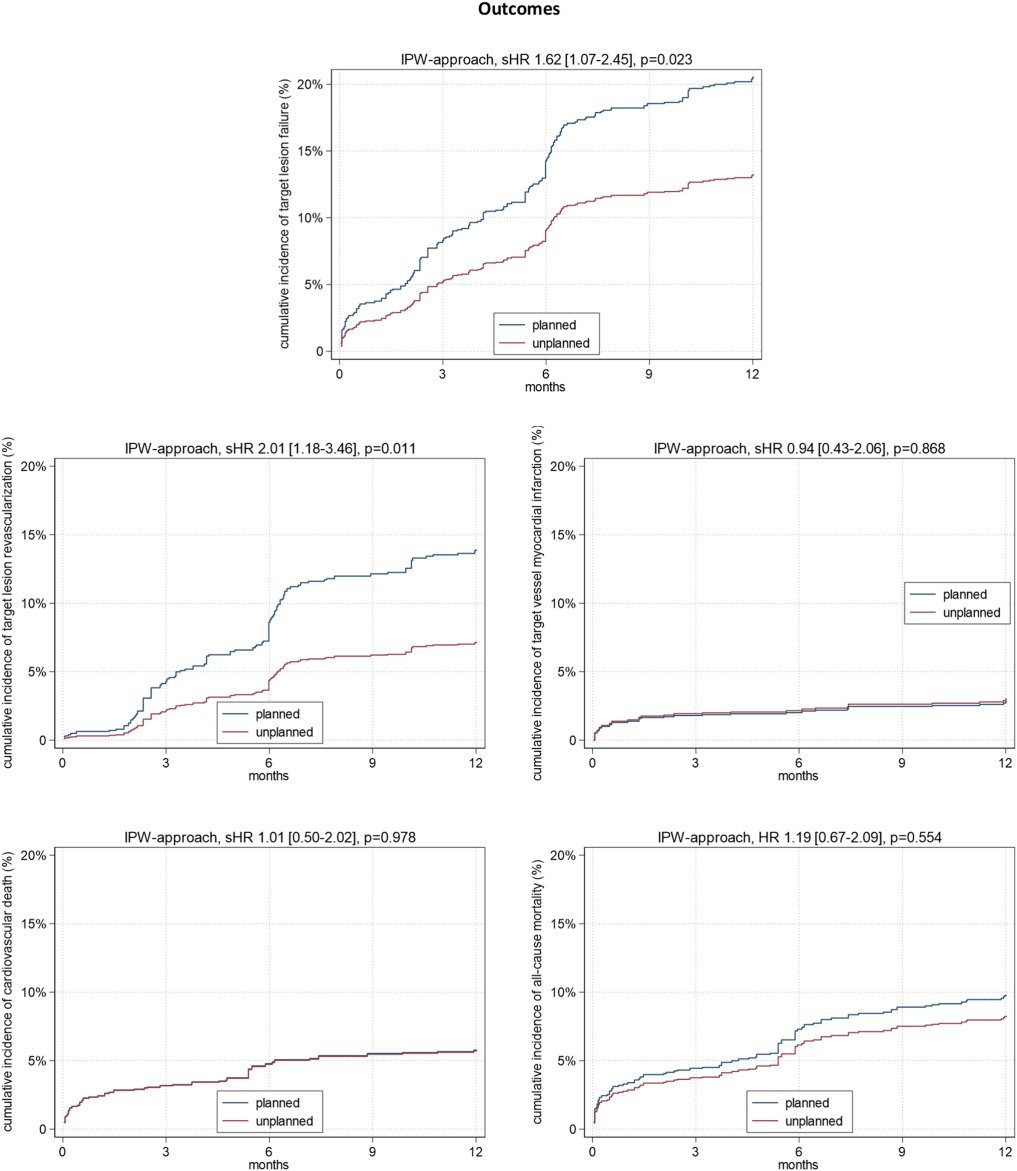
Weighted 1-year outcomes after planned versus unplanned rotational atherectomy. Predicted cumulative incidences (based on the weighted regression models) of the composite endpoint target lesion failure comprising target lesion revascularization, target vessel myocardial infarction, cardiovascular death and all-cause mortality for planned (blue) or unplanned (red) rotational atherectomy. Please note that the 883 vessels refer to 818 patients. In case of multiple interventions in different vessels of the same patient, many cases were conducted within the same date (*N = *32) or using the same technique (either planned or unplanned RA in both vessels, *N = *58). Cases with multiple interventions at different dates (*N = *33) or using different techniques (planned or unplanned RA, *N = *17) are also existent and making the analysis of all endpoints on the level of the vessel (rather than the patient level) eminent

The primary composite endpoint of target lesion failure (TLF) comprising cardiovascular death, target vessel myocardial infarction (TVMI), and ischemia driven target lesion revascularization (TLR) 1 year after the index procedure occurred in 18.5% after planned, and in 14.7% after unplanned RA (*p = *0.163), see Fig. 2. Weighted subdistribution hazard ratios (sHR) revealed a higher sHR for TLFs in the planned group (1.62 [1.07–2.45], *p = *0.023), which was driven by TLR (2.01 [1.18–3.46], *p = *0.011). Weighted subdistribution hazard ratios for target vessel myocardial infarction (0.94 [0.43–2.06], *p = *0.868), cardiovascular death (1.01 [0.50–2.02], *p = *0.978), and all-cause mortality (1.19 [0.67–2.09], *p = *0.554) did not differ between the groups. See Fig. 3 for predicted cumulative incidences based on the weighted regression models. Covariate balancing statistics are shown in Supplementary Table 1 indicating that our models adequately balance the covariates.

## Discussion

To the best of our knowledge, this is the largest study investigating patients undergoing either planned or unplanned RA as a lesion preparation technique for heavily calcified coronary lesions. The main finding is that unplanned RA was not associated with either a higher rate of periprocedural complications or worse 1-year outcome. However, minor procedural characteristics such as procedure length and contrast volume were favorable in planned procedures.

### Procedural characteristics

We observed an angiographic success rate of ≥ 99% in both groups, which is in line with data reported by Kawamato et al. [11]. However, other groups describe lower success rates for unplanned RA [10, 13]. Contrary to the report of other authors, we did not observe higher periprocedural complication rates upon an upfront futile attempt of PCI in unplanned RA procedures [9, 10, 13]. Coronary perforations in both groups were in the range of previously published prospective data [7, 16]. As previously shown by others [9–13], the use of contrast volume, fluoroscopy time and radiation exposure were significantly higher in the unplanned group.

### One-year follow-up

Event rates were in the range of published data [9–13]. Driven by a significantly higher hazard ratio for TLR, weighted hazard ratios revealed a higher risk of TLF for planned procedures. These findings are somewhat surprising, since baseline patient characteristics associated with an increased restenosis risk like diabetes, prior MI and prior PCI were favorable in planned RAs. However, calcification severity was more pronounced, and main left interventions were more than three times more frequent in the planned group, a finding known to be independently associated with adverse 1-year outcome [17]. Therefore, we performed inverse probability weighting for the primary endpoint for interventions in the left main coronary artery as a sensitivity analyses, which showed the similar unfavorable trend for planned procedures (Supplementary Fig. 1). Overall, the data published so far have been inconclusive including a meta-analysis that identified no significant differences in the longer-term outcomes between planned and unplanned rotational atherectomy [18]. The favorable outcome for unplanned procedures in our study confirmed the results of the smaller ROTATE multi-center registry [11].

### Clinical implications

Our data add evidence that futile conventional PCI may not worsen periprocedural and long-term outcomes of subsequent unplanned RA and would thus support previous recommendations for a predominant use of RA in bailout situations [19]. To this regard, the relatively low cross-over rates of ROTAXUS [6] and PREPARE-CALC [7] (12.5% and 16%, respectively) provide valid arguments for an initial attempt of conventional PCI even in heavily calcified coronary lesions. Nevertheless, many experienced interventionalists regard a planned approach as favorable when angiographic or intravascular imaging detect appropriate lesions [20] and the current AHA/ACC/SCAI guidelines on coronary revascularization include a class 2A recommendation for RA in such situations [21]. In line with this, RA was performed as an unplanned procedure in less than one half of RA procedures at our center (46.6%), which agrees with previously published rates of 27–63% for unplanned use of RA at other centers [9–13, 22, 23]

### Limitations

Saliently, this study has retrospective, observational and non-randomized character and was performed at a single center which includes substantial selection and operator biases. Thus, unknown confounders that could not be corrected for in the weighted analyses, may have biased planned RA procedures towards inferior outcomes. Particularly, periprocedural complications after RA strongly and inversely correlate with operator volume [24] and may, therefore, limit the generalizability of our results to high-volume centers. Further, we did not assess the impact of DAPT- or intravascular imaging strategies on outcome after RA, which were both potential confounders. Nevertheless, our data represent unselected real-world data for a high-risk, all-comers’ population and though no systematic angiographic follow-up was performed, no patient was lost to clinical follow-up.

## Conclusion

Our findings add evidence to the use of unplanned rotational atherectomy as a safe and efficacious strategy in cases of insufficient lesion preparation. Nevertheless, to our extent, the existing evidence is still too limited to condense indications for planned rotational atherectomy in heavily calcified coronary lesions. Hence, randomized and prospective trials are needed to evaluate a predominant use of rotational atherectomy as a bailout strategy in the future.

### Impact on daily practice

This study showed that unplanned rotational atherectomy upon a futile attempt of upfront conventional PCI can be safely performed and may not be inferior to planned RA in a high-volume center. Our data add evidence that RA can safely be reserved for lesions that prove untreatable by conventional means.

## Supplementary Information

Below is the link to the electronic supplementary material.

Supplementary file1 (TIF 592 kb)

Supplementary file2 (DOCX 72 kb)

## Data Availability

Data available on request from the authors.

## Abbreviations

MI: Myocardial infarction
PCI: Percutaneous coronary intervention
RA: Rotational atherectomy
TLF: Target lesion failure
TVR: Target vessel revascularization
