# Supplementary figures and images for: Planned versus unplanned rotational atherectomy for plaque modification in severely calcified coronary lesions
Source: Clin Res Cardiol. 2023 Mar 17;112(9):1252–62. doi: 10.1007/s00392-023-02176-6 (PMC10449691; doi:10.1007/s00392-023-02176-6)

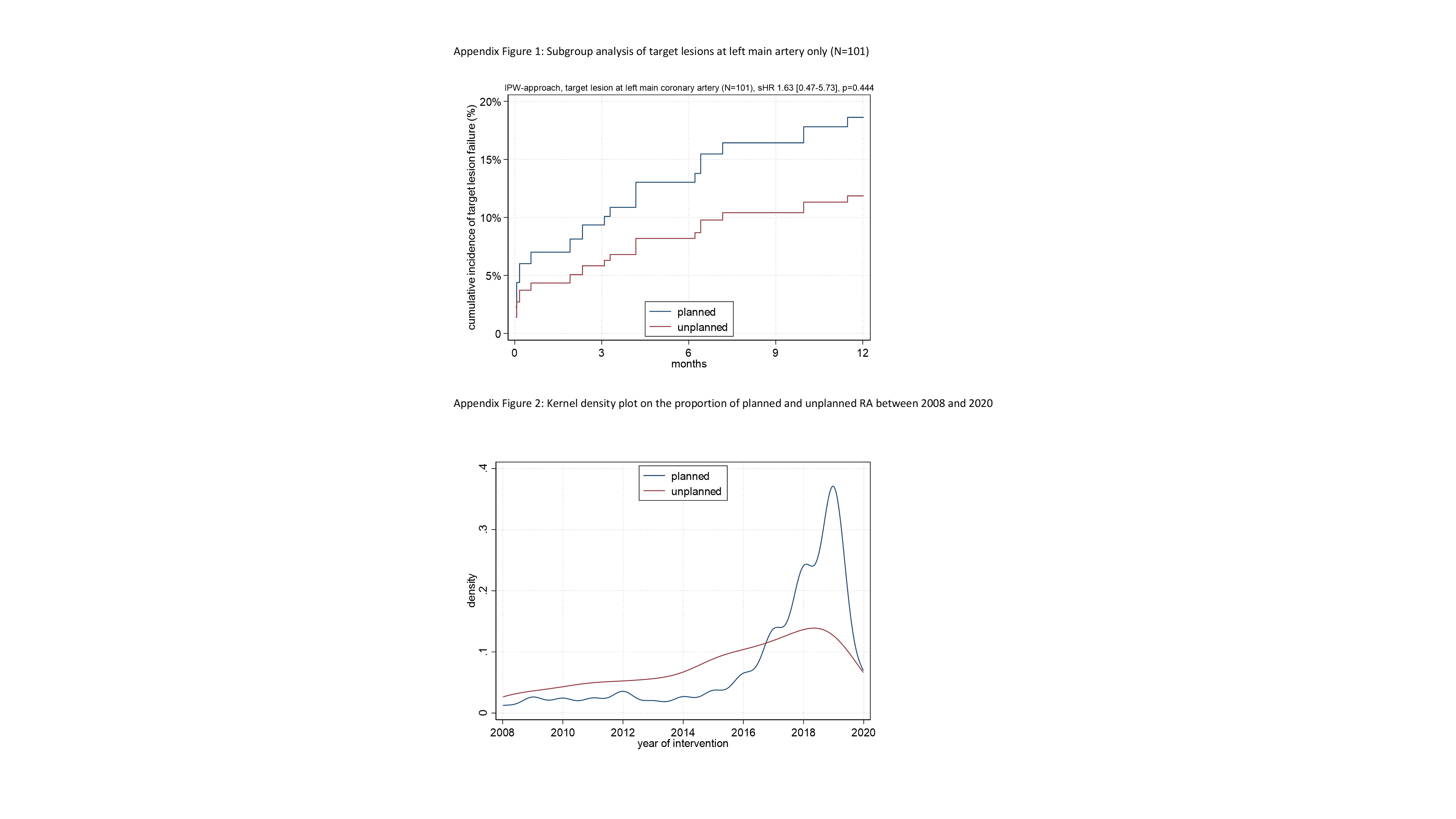

Supplement: Supplementary file 1 — Supplementary file1 (TIF 592 kb) [file 392_2023_2176_MOESM1_ESM.tif]
