# Supplementary material for: Planned versus unplanned rotational atherectomy for plaque modification in severely calcified coronary lesions
Source: Clin Res Cardiol. 2023 Mar 17;112(9):1252–62. doi: 10.1007/s00392-023-02176-6 (PMC10449691; doi:10.1007/s00392-023-02176-6)
Supplement: Supplementary file 2 — Supplementary file2 (DOCX 72 kb) [file 392_2023_2176_MOESM2_ESM.docx]

Appendix Table 1: Covariate balancing statistics for the IPW-approach

|  | Standardized differences | |
| --- | --- | --- |
|  | Raw | Weighted |
|  |  |  |
| Age | 0.333 | 0.023 |
| Male | 0.012 | 0.017 |
| Hypertension | -0.008 | -0.014 |
| Diabetes | -0.142 | 0.057 |
| Smoking | -0.073 | 0.041 |
| BMI | -0.149 | 0.032 |
| Family history of premature CAD | -0.046 | 0.007 |
| LDL-cholesterol | 0.031 | -0.010 |
| Creatinine | 0.101 | 0.024 |
| CRP | 0.057 | 0.005 |
| Hemoglobine | -0.091 | -0.018 |
| Thrombocyte | -0.020 | 0.065 |
| Prior MI | -0.235 | -0.048 |
| Prior PCI | -0.160 | 0.014 |
| Prior CABG | -0.065 | -0.041 |
| LVEF | -0.049 | 0.028 |
| Coronary artery disease | 0.184 | 0.017 |
| Target lesion | -0.437 | 0.038 |
| Grade of calcification | 0.132 | 0.007 |
| Angulation of the lesion | -0.162 | -0.028 |
| Lesion length | -0.039 | 0.027 |
| Ostial lesions | 0.353 | -0.012 |
| Excentric lesions | -0.352 | -0.031 |
| Tortuos lesions | -0.104 | 0.069 |
| Chronic total occlusions | -0.294 | 0.040 |
| Lesions treated | 0.363 | 0.019 |
| Device | 0.502 | -0.017 |
| Burr size | 0.499 | -0.041 |
| Sheath size | 0.374 | 0.002 |
| Balloon diameter | 0.074 | -0.005 |
| Peak inflation pressure | -0.147 | -0.006 |
| Max. stent diameter | 0.127 | 0.033 |
| Peak pressure at stent delivery | -0.069 | -0.022 |
| Stent length | -0.194 | 0.061 |
| Use of an OPN-balloon | -0.356 | 0.060 |
| Number of stents implanted | 0.152 | 0.019 |
| Stents | 0.090 | 0.028 |
| Stent polymer coating | 0.191 | -0.009 |
| Angiographic success | 0.003 | 0.013 |
| Radiation exposure | -0.428 | 0.026 |
| Fluoroscopy time | -0.450 | 0.015 |
| Contrast volume | -0.281 | 0.006 |
